# Impact of parameter control on the performance of APSO and PSO algorithms for the CSTHTS problem: An improvement in algorithmic structure and results

**DOI:** 10.1371/journal.pone.0261562

**Published:** 2021-12-17

**Authors:** Muhammad Ahmad Iqbal, Muhammad Salman Fakhar, Syed Abdul Rahman Kashif, Rehan Naeem, Akhtar Rasool

**Affiliations:** 1 Department of Electrical Engineering, University of Engineering and Technology, Lahore, Punjab, Pakistan; 2 Department of Electrical Engineering, Sharif College of Engineering and Technology, Lahore, Punjab, Pakistan; Northeast Electric Power University, CHINA

## Abstract

Cascaded Short Term Hydro-Thermal Scheduling problem (CSTHTS) is a single objective, non-linear multi-modal or convex (depending upon the cost function of thermal generation) type of Short Term Hydro-Thermal Scheduling (STHTS), having complex hydel constraints. It has been solved by many metaheuristic optimization algorithms, as found in the literature. Recently, the authors have published the best-achieved results of the CSTHTS problem having quadratic fuel cost function of thermal generation using an improved variant of the Accelerated PSO (APSO) algorithm, as compared to the other previously implemented algorithms. This article discusses and presents further improvement in the results obtained by both improved variants of APSO and PSO algorithms, implemented on the CSTHTS problem.

## Introduction

The CSTHTS is a type of highly multi-modal, multi-dimensional, non-linear, and non-convex optimization problem which deals with the economic dispatch of power among hydel and thermal generating units. It can be modeled in generic form using the Eqs 1–9, as suggested in reference [1].

$$\begin{array}{c} min\left(f\right)=\sum_{m=1}^{N}\sum_{i=1}^{T}n_{m}F_{\left(m,i\right)} \end{array}$$ (1)

where, *n*_*m*_ is the number of hours in the scheduling period ‘*m*’ and *F*_(*m*,*i*)_ is the per hour cost of the thermal generating unit. Eq 1 presents the main objective function of the CSTHTS problem, i.e., to minimize the total cost of the scheduling of thermal generators.

$$\begin{array}{c} \sum_{m=1}^{N}(\sum_{i=1}^{N_{s}}P_{th_{i,m}}+\sum_{j=1}^{N_{h}}P_{hyd_{j,m}})=P_{d}+P_{l} \end{array}$$ (2)

The equality constraint of the CSTHTS problem is shown by Eq 2, which assures that the sum of powers generated by both thermal $P_{th_{i,m}}$ and hydel $P_{hyd_{j,m}}$ plants are equal to the sum of demand power *P*_*d*_ and transmission losses *P*_*l*_ of the power system for a total time of *N* scheduling intervals. Where, *N*_*s*_ is the total number of thermal plants, *N*_*h*_ is the total number of water reservoirs.

$$\begin{array}{c} P_{hyd_{j,m}}=f\left(V_{hyd_{j,m}},Q_{hyd_{j,m}}\right) \end{array}$$ (3)

Eq 3 presents hydel power as a function of the volume $V_{hyd_{j,m}}$ and water discharge rate $Q_{hyd_{j,m}}$ at scheduling interval ‘*m*’ of *j*^*th*^ reservoir.

$$\begin{array}{c} P_{th_{i}}^{min}\leq P_{th_{i,m}}\leq P_{th_{i}}^{max} \end{array}$$ (4)

$$\begin{array}{c} P_{hyd_{j}}^{min}\leq P_{hyd_{j,m}}\leq P_{hyd_{j}}^{max} \end{array}$$ (5)

Eqs 4 and 5 give the bounded power limits for the *i*^*th*^ thermal and the *j*^*th*^ hydel unit, respectively at the *m*^*th*^ scheduling interval. Where, $P_{th_{i}}^{min}$ and $P_{th_{i}}^{max}$ are the minimum and maximum allowable value of thermal generation for the *i*^*th*^ thermal unit, respectively. And $P_{hyd_{j}}^{min}$ and $P_{hyd_{j}}^{max}$ are the minimum and maximum allowable value of hydel generation for the *j*^*th*^ water reservoir, respectively.

$$\begin{array}{c} V_{hyd_{j}}^{min}\leq V_{hyd_{j,m}}\leq V_{hyd_{j}}^{max} \end{array}$$ (6)

$$\begin{array}{c} Q_{hyd_{j}}^{min}\leq Q_{hyd_{j,m}}\leq Q_{hyd_{j}}^{max} \end{array}$$ (7)

Eqs 6 and 7 are related to the operation of the water reservoir. Where, $V_{hyd_{j}}^{min}$ and $V_{hyd_{j}}^{max}$ are the minimum and maximum allowable value of the volume of the *j*^*th*^ reservoir at *m*^*th*^ scheduling interval and $Q_{hyd_{j}}^{min}$ and $Q_{hyd_{j}}^{max}$ are the minimum and maximum allowable value of the discharge rate of the *j*^*th*^ reservoir at *m*^*th*^ scheduling interval.

$$\begin{array}{c} \sum_{m=1}^{N}Q_{hyd_{j,m}}=Q_{hyd_{j,total}} \end{array}$$ (8)

Eq 8 gives the allowed value of the water discharged $Q_{hyd_{j,total}}$ by the *j*^*th*^ reservoir for a total time of *N* scheduling intervals.

$$\begin{array}{c} \begin{array}{cc} V_{hyd_{j,(m+1)}}=\; & V_{hyd_{j,m}}+I_{hyd_{j,m}}-Q_{hyd_{j,m}}-S_{hyd_{j,m}}+\\ & \sum_{a=1}^{R_{u,j}}(Q_{{hyd}_{a,(m-t)}}+S_{{hyd}_{a,(m-t)}}) \end{array} \end{array}$$ (9)

The continuity equation balances the discharges and reservoir volume and is shown by Eq 9. Where, ‘*m*’ is the scheduling hour, ‘*j*’ is the reservoir available for hydel generation, $I_{hyd_{j,m}}$ is the inflow of the reservoir, $S_{{hyd}_{a,(m-t)}}$ is the spillage of the reservoir, *R*_*u*,*j*_ are the upstream reservoirs present for the *j*^*th*^ reservoir and ‘*t*’ is the water transport delay from reservoir ‘*a*’ to reservoir ‘*j*’.

$$\begin{array}{c} F_{\left(m,i\right)}=a+bP_{th_{\left(i,m\right)}}+cP_{th_{\left(i,m\right)}}^{2} \end{array}$$ (10)

The cost *F*_(*m*,*i*)_ of *i*^*th*^ thermal unit at *m*^*th*^ scheduling interval is the function of the power of the thermal generator which is the function of the fuel cost. The relation of cost and thermal power is given by Eq 10, which is a quadratic function (convex) of thermal power and ‘*a*’, ‘*b*’ and ‘*c*’ are the coefficients of the scheduling equation.

Eq 10 can be of higher order depending upon the type of thermal generator, causing an increase in the nonlinearity of the objective function. The CSTHTS problem is a scheduling problem having many scheduling intervals and each interval is treated as one dimension of the problem, which makes the CSTHTS highly multi-modal, i.e., a problem with an objective function having multiple peaks.

The test system of the CSTHTS problem selected in this article is a twenty-four hours long scheduling problem having one thermal unit and four hydel units for power generation. It is solved without considering the valve-point effect of thermal units and transmission losses. Data for the cost coefficients of the thermal unit and power production coefficients of hydro units are taken from reference [2].

It can be misinterpreted as a convex problem by just looking at the thermal fuel cost function (which is quadratic) as it is the main objective function but as a whole when this objective function is taken along with all the time coupling hydel and thermal constraints for twenty-four scheduling intervals it becomes multi-modal and non-convex because the thermal power generated depends upon the hydel generation, as mentioned in Eq 2. The function used to calculate hydel generation, as mentioned in Eq 3 is non-convex in nature as it contains multiplication terms of volume and discharge rate of the reservoir generating power to meet the load demand. The multiplication of two variables is a non-convex function according to reference [3]. This article discusses a highly complex, multi-modal, and non-convex case of the CSTHTS problem having quadratic thermal fuel cost function, benchmark case of the CSTHTS problem.

The CSTHTS problem, according to reference [1], is concerned with the combined economic dispatch of a chain of numerous water reservoirs located on the same stream in sequence, i.e., one reservoir-based hydel power plant is downhill from the other reservoir-based hydel power plant. There may be numerous thermal power plants in such problems, although they are normally treated as equivalent thermal generating units. if *j* > 1, the CSTHTS problem is defined by equality and non-equality equations given in Eqs 1–9.

The genetic algorithm has been implemented in reference [2] to optimize the CSTHTS problem. Reference [4], has implemented the dynamic search space squeezing based APSO on the STHTS problem. Reference [5], presents APSO and firefly algorithm on the hybrid case of STHTS problem which incorporates the effects of adding solar cells to the conventional grid. Dynamically search space squeezing based firefly algorithm has been implemented on the hybrid case of the STHTS problem in reference [6]. References [4–6], have shown that the superiority of one metaheuristic algorithm over the other should be made by performing the proper statistical tests, not by just comparing their achieved optimal solution. Three variants of evolutionary programming algorithms have been implemented to solve the CSTHTS problem, known as FEP, IFEP, and CEP, in reference [7]. The particle swarm optimization (PSO) algorithm has been implemented in reference [8] to solve the CSTHTS problem. The CSTHTS problem has been solved by adaptive and modified adaptive PSO in reference [9]. The differential evolution algorithm has been implemented in reference [10] to optimize the CSTHTS problem. The real coded genetic algorithm has been used in reference [11] to find the optimal solution to the CSTHTS problem. The IPSO, EPSO, and EGA algorithms have been implemented in reference [12] to optimize the CSTHTS problem. The modified differential evolution algorithm has been used in reference [13] to solve the CSTHTS problem. The teaching learning-based algorithm has been used in reference [14] to find the approximate global optimum solution of the CSTHTS problem. Reference [15], has used an artificial fish swarm and a real coded genetic hybrid algorithm to solve the CSTHTS problem. Reference [16], implemented PSO-ALNS algorithm, moth-flame optimization, grey wolf optimization, and a combination of grey wolf and dragonfly algorithm to obtain the global optimum solution of the CSTHTS problem. Reference [17], showed that the small population-based PSO to be the best algorithm to find the optimal robust solution of the CSTHTS problem.

A detailed review of the CSTHTS problem solved by different conventional and metaheuristic algorithms in past was presented in reference [18]. The improved PSO with adaptive cognitive and social components has been implemented in reference [19], to optimize the CSTHTS problem. In Reference [19], the algorithm has two update equations which increase the complexity of implementing it on large-scale optimization problems. A novel PSO technique has been implemented in reference [20], which used the concept of adaptive inertia weight constant to solve the CSTHTS problem. In this technique, the social and cognitive coefficients were not considered. The diversified PSO technique has been implemented in reference [21], to find the approximate global optimum solution of the CSTHTS problem. However, this suggested technique depends upon the optimal selection of the population size.

PSO and APSO in their canonical versions can have an issue of premature convergence. One of the most recent methods to cope with the issue of premature convergence is to dynamically utilize extreme learning machine mechanisms along with adopting mutation strategy to have a dynamic update of the particles, as presented and proved by reference [22]. However, it has been carefully observed by the authors of this article that adopting mutation strategies along with extreme learning machines is not required in the newly proposed variants of improved APSO and improved PSO, while adopting the newly suggested constraint handling technique. The tuning of *α* and *β* coefficients in APSO variants and the acceleration and inertia coefficients in improved PSO makes sure that the particles do not get stuck to local optima, by improving the global search ability of the particles at the initial stages of the iterative process. However, for extremely high multi-modal and non-convex optimization problems, premature convergences can be avoided by utilizing the techniques suggested by reference [22].

According to no free lunch theorems presented in reference [23], if an algorithm performs well when applied to an optimization problem, it does not guarantee that it will perform well on other types of optimization problems. Therefore, it is required to find a suitable optimization algorithm for each type of optimization problem, if taken individually. In this article, a highly complex and multi-modal case of the CSTHTS problem is solved by implementing sixteen variants of the APSO algorithm and one variant of the PSO algorithm. The results obtained have been compared with previously found optimal solutions of the selected test case by other conventional and metaheuristic algorithms, reported in the literature.

In this article, the CSTHTS case has been solved by using the newly proposed sixteen improved variants of APSO and an improved variant of PSO along with a newly proposed constraint handling technique as an original contribution. It has been presented that the PSO variant has given so far the best results of the CSTHTS case, as compared to the previously reported results of the implementation of other algorithms, as found in literature [24]. Reference [24], previously published by the authors of this article, gave the so far best-achieved results of the selected test case of the CSTHTS problem. However, there were two motivations to further improve the algorithms in search of finding better solutions. Firstly, the results were achieved in reference [24] used a larger number of particles (5000) and a large number of iterations (5000), having a high value of standard deviation, and the average time took to simulate one trial was 243 seconds. Therefore, it was needed to further improve the algorithms so that the results can be achieved using a smaller number of particles, having a lower value of standard deviation, and in lesser computational time. Secondly, it was needed to further investigate that if using the new modifications in APSO and PSO algorithms can help to achieve further improvement in results. The findings of this new experiment on the CSTHTS case by designing and implementing the newly proposed variants of PSO and APSO have been presented in this article. It has been shown that both improved variants of APSO and PSO helped to achieve better results in a very less computational time as compared to reference [24]. Though the improved PSO was able to find the nearest approximation to the global optimum solution, however, for repeated trials, the standard deviation of the results achieved by the improved PSO variant remained very high. The sixteen improved variants of APSO then served the purpose of keeping the standard deviation value lower while achieving almost near results to that achieved by the improved PSO variant for the best trial. A comparative analysis of the results achieved by the improved PSO variant is made with the best among the sixteen variants of improved APSO using statistical tests and is presented in this article.

The paper is organized as follows: Section 2 describes the algorithmic structure of the sixteen variants of APSO along with presenting the pseudo-code for the newly proposed constraint handling technique for the CSTHTS case, Section 3 presents the algorithmic structure of the improved PSO variant, Section 4 describes the results of the implementations of improved PSO variant and sixteen variants of improved APSO on the CSTHTS case, Section 5 gives the discussion on the results achieved, and finally, section 6 concludes the paper.

## Improvements of APSO algorithm

The APSO algorithm, presented in reference [23], is a very promising version of the canonical PSO. When compared to the original PSO algorithm, it can be witnessed that it has a single update equation as described by Eq 11 for the particles without requiring the velocity update equation, making it simple to apply to the optimization problem.

$$\begin{array}{c} x_{i}^{t+1}=\left(1-\beta \right)x_{i}^{t}+\beta g+\alpha \epsilon \end{array}$$ (11)

where, the typical values of *α* and *β* are usually taken as 0.2 and 0.5 respectively in the canonical version.

In this article, the authors have applied another modification in the improved APSO algorithm, that was implemented on the CSTHTS case in reference [24]. The improved variant has a single-step update equation without utilizing the velocity update equation as given in Eq 12.

$$\begin{array}{c} x_{i}^{t+1}=\left(1-\beta \left(t\right)\right)p_{i}^{t}+\beta \left(t\right)g^{t}+\alpha \left(t\right)R_{i}^{t} \end{array}$$ (12)

The values of *α* and *β* should be in between 0 and 1 and their most effective range of performance is in between the maximum and minimum limits mentioned in reference [23]. Increasing the values of *α* and *β* greater than 1 causes the solution of the APSO algorithm to diverge. The values of *α* and *β* are normally taken as fixed in the canonical form, according to reference [23]. The improvements are made in the canonical APSO by modifying the *α* and *β* coefficients. In reference [24], *α* is varied from 0.6 to 0.2 and *β* is varied from 0.7 to 0.1. The authors of this paper add further improvement in the improved version of the APSO algorithm of reference [24] and suggest sixteen different variants of improved APSO in terms of *α* and *β* parameter control. The results achieved are significantly better than the results of reference [24]. Another improvement in Eq 11, is using the local best $p_{i}^{t}$ of each particle at any iteration *k*, instead of the particle’s current position at time *t*, as suggested in reference [25].

Where, $R_{i}^{t}$ is an *N* × *d* dimensional matrix of uniform random numbers given in reference [25], where *N* is the particles generated and *d* is the total scheduling intervals of the CSTHTS problem. Randomization of the APSO algorithm is increased by using higher values of *α* to avoid premature convergence to local optima. When the APSO algorithm progresses to the end of iterations, during this time particles are converging towards the global optimum solution avoiding the local optimum solutions. So, it is required that the particles do not diverge (oscillates) from the global optimum solution. Therefore, *α* is decreased from a higher value to a lower value as the algorithm proceeds from the first iteration to the last iteration. This decay in the value of *α* may be sinusoidal, linear, quadratic, or exponential depending upon the type of optimization problem under consideration. It was observed that the linear decay in the value of *α* gives a good performance of the APSO algorithm applied to the CSTHTS problem.

The value of *β* decides the weight given to the particle global best and local best of the iteration. It can be a constant as well as varying value. It has been observed that different chaotic maps applied to vary the value of *β* gives a good performance of the APSO algorithm instead of making it fixed. This will help to keep the solution space diversity alive, similar to the case of the levy flight concept in cuckoo search and firefly algorithm.

To conclude, at the starting point of the APSO algorithm, the *α* value should be high to increase the particles exploration. However, step reduction of the value of *α* should be made as the APSO algorithm start proceeding towards the end to make the APSO algorithm converge at a good approximate of the global optimum solution. Higher values of *β* allow particles to have a greater influence from the global best of each iteration and the lower value to have more influence from the local best of each iteration. Brilliant results have been achieved by making these modifications in the canonical APSO algorithm.

### Variants of APSO

Parameter control setting of APSO for sixteen different chaotic maps has been implemented on the test case of the CSTHTS problem, which gives improved results compared with reference [24], among all the implemented chaotic maps, variant-16 gives the minimum cost. It is important to mention that a newly proposed constraint handling technique has been incorporated by the authors while implementing all the variants of APSO. The pseudo-code of this constraint handling technique has been presented in Table 1. Each variant is simulated for 50 trials having 5050 iterations and population size of 600. Details of sixteen chaotic maps implemented are given as follows.

**Table 1:** Pseudocode for newly proposed constraint handling technique.

**If** (Qi,d <Qmin)
Find difference = Qmin—Qi,d
Set Qi,d = Qmin (hard limit)
**End If**
**For** n = 23 to 1
Adjust the difference in 23 entries while keeping them in limits. Starting from 23rd entry to 1st entry.
**End For**
**Else If** (Qi,d >Qmax)
Find difference = Qi,d– Qmax
Set Qi,d = Qmax (hard limit)
**For** n = 1 to 23
Adjust the difference in 23 entries while keeping them in limits. Starting from 1st entry to 23rd entry.
**End For**

#### Variant 1

In this variant, *α* is sinusoidally decreased up to 1/4 of wavelength (90°) from 0.81 to 0.62. Values of *α*_*max*_ and *α*_*min*_ in this variant are 0.81 and 0.62, respectively as shown in Eq 13. The value of *β* is linearly decreased from 0.81 to 0.62. Values of *β*_*max*_ and *β*_*min*_ in this variant are 0.81 and 0.62, respectively as shown in Eq 14.

$$\begin{array}{c} \alpha =\alpha_{min}+\left(\alpha_{max}-\alpha_{min}\right)\times \text{cos}(\frac{\pi }{2}\times \frac{Iteration_{current}}{Iteration_{total}}) \end{array}$$ (13)

$$\begin{array}{c} \beta =\beta_{max}-(\left(\beta_{max}-\beta_{min}\right)\times \frac{Iteration_{current}}{Iteration_{total}}) \end{array}$$ (14)

#### Variant 2

In this variant, *α* is linearly decreased from 0.81 to 0.62. Values of *α*_*max*_ and *α*_*min*_ in this variant are 0.81 and 0.62, respectively as shown in Eq 15. The value of *β* is sinusoidally decreased up to 1/2 of wavelength (180°) from 0.81 to 0.62. Values of *β*_*max*_ and *β*_*min*_ in this variant are 0.81 and 0.715, respectively as shown in Eq 16.

$$\begin{array}{c} \alpha =\alpha_{max}-(\left(\alpha_{max}-\alpha_{min}\right)\times \frac{Iteration_{current}}{Iteration_{total}}) \end{array}$$ (15)

$$\begin{array}{c} \beta =\beta_{min}+\left(\beta_{max}-\beta_{min}\right)\times \text{cos}(\pi \times \frac{Iteration_{current}}{Iteration_{total}}) \end{array}$$ (16)

#### Variant 3

In this variant, *α* and *β* both are sinusoidally decreased up to 1/4 of wavelength (90°) from 0.81 to 0.62. Values of *α*_*max*_, *α*_*min*_, *β*_*max*_ and *β*_*min*_ in this variant are 0.81, 0.62, 0.81 and 0.62, respectively as shown in Eqs 17 and 18.

$$\begin{array}{c} \alpha =\alpha_{min}+\left(\alpha_{max}-\alpha_{min}\right)\times \text{cos}(\frac{\pi }{2}\times \frac{Iteration_{current}}{Iteration_{total}}) \end{array}$$ (17)

$$\begin{array}{c} \beta =\beta_{min}+\left(\beta_{max}-\beta_{min}\right)\times \text{cos}(\frac{\pi }{2}\times \frac{Iteration_{current}}{Iteration_{total}}) \end{array}$$ (18)

#### Variant 4

In this variant, *α* and *β* are linearly decreased from 0.81 to 0.62. Values of *α*_*max*_, *α*_*min*_, *β*_*max*_ and *β*_*min*_ in this variant are 0.81, 0.62, 0.81 and 0.62, respectively as shown in Eqs 19 and 20.

$$\begin{array}{c} \alpha =\alpha_{max}-(\left(\alpha_{max}-\alpha_{min}\right)\times \frac{Iteration_{current}}{Iteration_{total}}) \end{array}$$ (19)

$$\begin{array}{c} \beta =\beta_{max}-(\left(\beta_{max}-\beta_{min}\right)\times \frac{Iteration_{current}}{Iteration_{total}}) \end{array}$$ (20)

#### Variant 5

In this variant, *α* and *β* both are sinusoidally decreased up to 1/2 of wavelength (180°) from 0.81 to 0.62. Values of *α*_*max*_, *α*_*min*_, *β*_*max*_ and *β*_*min*_ in this variant are 0.81, 0.715, 0.81 and 0.715, respectively as shown in Eqs 21 and 22.

$$\begin{array}{c} \alpha =\alpha_{min}+\left(\alpha_{max}-\alpha_{min}\right)\times \text{cos}(\pi \times \frac{Iteration_{current}}{Iteration_{total}}) \end{array}$$ (21)

$$\begin{array}{c} \beta =\beta_{min}+\left(\beta_{max}-\beta_{min}\right)\times \text{cos}(\pi \times \frac{Iteration_{current}}{Iteration_{total}}) \end{array}$$ (22)

#### Variant 6

In this variant, *α* is sinusoidally decreased up to 1/2 of wavelength (180°) from 0.81 to 0.62. Values of *α*_*max*_ and *α*_*min*_ in this variant are 0.81 and 0.715, respectively as shown in Eq 23. The value of *β* is linearly decreased from 0.81 to 0.62. Values of *β*_*max*_ and *β*_*min*_ in this variant are 0.81 and 0.62, respectively as shown in Eq 24.

$$\begin{array}{c} \alpha =\alpha_{min}+\left(\alpha_{max}-\alpha_{min}\right)\times \text{cos}(\pi \times \frac{Iteration_{current}}{Iteration_{total}}) \end{array}$$ (23)

$$\begin{array}{c} \beta =\beta_{max}-(\left(\beta_{max}-\beta_{min}\right)\times \frac{Iteration_{current}}{Iteration_{total}}) \end{array}$$ (24)

#### Variant 7

In this variant, *α* is sinusoidally decreased up to 1/4 of wavelength (90°) from 0.81 to 0.62. Values of *α*_*max*_ and *α*_*min*_ in this variant are 0.81 and 0.62, respectively as shown in Eq 25. The value of *β* is fixed at 0.715.

$$\begin{array}{c} \alpha =\alpha_{min}+\left(\alpha_{max}-\alpha_{min}\right)\times \text{cos}(\frac{\pi }{2}\times \frac{Iteration_{current}}{Iteration_{total}}) \end{array}$$ (25)

#### Variant 8

In this variant, *α* is linearly decreased from 0.81 to 0.62. Values of *α*_*max*_ and *α*_*min*_ in this variant are 0.81 and 0.62, respectively as shown in Eq 26. The value of *β* is fixed at 0.715.

$$\begin{array}{c} \alpha =\alpha_{max}-(\left(\alpha_{max}-\alpha_{min}\right)\times \frac{Iteration_{current}}{Iteration_{total}}) \end{array}$$ (26)

#### Variant 9

In this variant, *α* is sinusoidally decreased up to 1/2 of wavelength (180°) from 0.81 to 0.62. Values of *α*_*max*_ and *α*_*min*_ in this variant are 0.81 and 0.715, respectively as shown in Eq 27. The value of *β* is fixed at 0.715.

$$\begin{array}{c} \alpha =\alpha_{min}+\left(\alpha_{max}-\alpha_{min}\right)\times \text{cos}(\pi \times \frac{Iteration_{current}}{Iteration_{total}}) \end{array}$$ (27)

#### Variant 10

In this variant, *α* is sinusoidally decreased up to 1/4 of wavelength (90°) from 0.81 to 0.62. Values of *α*_*max*_ and *α*_*min*_ in this variant are 0.81 and 0.62, respectively as shown in Eq 28. The value of *β* is linearly increased from 0.62 to 0.81. Values of *β*_*max*_ and *β*_*min*_ in this variant are 0.81 and 0.62, respectively as shown in Eq 29.

$$\begin{array}{c} \alpha =\alpha_{min}+\left(\alpha_{max}-\alpha_{min}\right)\times \text{cos}(\frac{\pi }{2}\times \frac{Iteration_{current}}{Iteration_{total}}) \end{array}$$ (28)

$$\begin{array}{c} \beta =\beta_{min}+(\left(\beta_{max}-\beta_{min}\right)\times \frac{Iteration_{current}}{Iteration_{total}}) \end{array}$$ (29)

#### Variant 11

In this variant, *α* is sinusoidally decreased up to 1/2 of wavelength (180°) from 0.81 to 0.62. Values of *α*_*max*_ and *α*_*min*_ in this variant are 0.81 and 0.715, respectively as shown in Eq 30. The value of *β* is linearly increased from 0.62 to 0.81. Values of *β*_*max*_ and *β*_*min*_ in this variant are 0.81 and 0.62, respectively as shown in Eq 31.

$$\begin{array}{c} \alpha =\alpha_{min}+\left(\alpha_{max}-\alpha_{min}\right)\times \text{cos}(\pi \times \frac{Iteration_{current}}{Iteration_{total}}) \end{array}$$ (30)

$$\begin{array}{c} \beta =\beta_{min}+(\left(\beta_{max}-\beta_{min}\right)\times \frac{Iteration_{current}}{Iteration_{total}}) \end{array}$$ (31)

#### Variant 12

In this variant, *α* is linearly decreased from 0.81 to 0.62. Values of *α*_*max*_ and *α*_*min*_ in this variant are 0.81 and 0.62, respectively as shown in Eq 32. The value of *β* is sinusoidally decreased up to 1/4 of wavelength (90°) from 0.81 to 0.62. Values of *β*_*max*_ and *β*_*min*_ in this variant are 0.81 and 0.62, respectively as shown in Eq 33.

$$\begin{array}{c} \alpha =\alpha_{max}-(\left(\alpha_{max}-\alpha_{min}\right)\times \frac{Iteration_{current}}{Iteration_{total}}) \end{array}$$ (32)

$$\begin{array}{c} \beta =\beta_{min}+\left(\beta_{max}-\beta_{min}\right)\times \text{cos}(\frac{\pi }{2}\times \frac{Iteration_{current}}{Iteration_{total}}) \end{array}$$ (33)

#### Variant 13

In this variant, *α* is sinusoidally decreased up to 1/2 of wavelength (180°) from 0.81 to 0.62. Values of *α*_*max*_ and *α*_*min*_ in this variant are 0.81 and 0.715, respectively as shown in Eq 34. The value of *β* is sinusoidally increased up to 1/2 of wavelength (180°) from 0.62 to 0.81. Values of *β*_*max*_ and *β*_*min*_ in this variant are 0.81 and 0.715, respectively as shown in Eq 35.

$$\begin{array}{c} \alpha =\alpha_{min}+\left(\alpha_{max}-\alpha_{min}\right)\times \text{cos}(\pi \times \frac{Iteration_{current}}{Iteration_{total}}) \end{array}$$ (34)

$$\begin{array}{c} \beta =\beta_{min}-\left(\beta_{max}-\beta_{min}\right)\times \text{cos}(\pi \times \frac{Iteration_{current}}{Iteration_{total}}) \end{array}$$ (35)

#### Variant 14

In this variant, *α* is linearly decreased from 0.81 to 0.62. Values of *α*_*max*_ and *α*_*min*_ in this variant are 0.81 and 0.62, respectively as shown in Eq 36. The value of *β* is sinusoidally increased up to 1/2 of wavelength (180°) from 0.81 to 0.62. Values of *β*_*max*_ and *β*_*min*_ in this variant are 0.81 and 0.715, respectively as shown in Eq 37.

$$\begin{array}{c} \alpha =\alpha_{max}-(\left(\alpha_{max}-\alpha_{min}\right)\times \frac{Iteration_{current}}{Iteration_{total}}) \end{array}$$ (36)

$$\begin{array}{c} \beta =\beta_{min}-\left(\beta_{max}-\beta_{min}\right)\times \text{cos}(\pi \times \frac{Iteration_{current}}{Iteration_{total}}) \end{array}$$ (37)

#### Variant 15

In this variant, *α* is sinusoidally decreased up to 1/4 of wavelength (90°) from 0.81 to 0.62. Values of *α*_*max*_ and *α*_*min*_ in this variant are 0.81 and 0.62, respectively as shown in Eq 38. The value of *β* is sinusoidally increased up to 1/4 of wavelength (90°) from 0.62 to 0.81. Values of *β*_*max*_ and *β*_*min*_ in this variant are 0.81 and 0.62, respectively as shown in Eq 39.

$$\begin{array}{c} \alpha =\alpha_{min}+\left(\alpha_{max}-\alpha_{min}\right)\times \text{cos}(\frac{\pi }{2}\times \frac{Iteration_{current}}{Iteration_{total}}) \end{array}$$ (38)

$$\begin{array}{c} \beta =\beta_{min}+\left(\beta_{max}-\beta_{min}\right)\times \text{sin}(\frac{\pi }{2}\times \frac{Iteration_{current}}{Iteration_{total}}) \end{array}$$ (39)

#### Variant 16

In this variant, *α* is linearly decreased from 0.81 to 0.62. Values of *α*_*max*_ and *α*_*min*_ in this variant are 0.81 and 0.62, respectively as shown in Eq 40. The value of *β* is sinusoidally increased up to 1/4 of wavelength (90°) from 0.81 to 0.62. Values of *β*_*max*_ and *β*_*min*_ in this variant are 0.81 and 0.62, respectively as shown in Eq 41. This gives the same variant as was utilized in reference [24], however, the minimum and maximum values of tuning parameters *α* and *β* play a significant role in improving the results for the CSTHTS problem. Moreover, by using these values of *α* and *β*, the required number of particles is reduced to 600, as compared to 5000 particles used in reference [24], which is a significant computational improvement. The computational time of simulating one trial of this variant is 45 seconds.

$$\begin{array}{c} \alpha =\alpha_{max}-(\left(\alpha_{max}-\alpha_{min}\right)\times \frac{Iteration_{current}}{Iteration_{total}}) \end{array}$$ (40)

$$\begin{array}{c} \beta =\beta_{min}+\left(\beta_{max}-\beta_{min}\right)\times \text{sin}(\frac{\pi }{2}\times \frac{Iteration_{current}}{Iteration_{total}}) \end{array}$$ (41)

## Improved PSO algorithm

The particle swarm optimization (PSO) in its canonical form has been a brilliant algorithm used for solving several optimization problems as found in the literature, according to [18]. However, the PSO algorithm in its canonical form was implemented in reference [26] on the CSTHTS case, and the results reported were under-rated. It has been observed that the constraint handling technique used in the reference [26] was the penalty factor approach. By using the newly proposed constraint handling technique of Table 1, PSO achieves markedly improved results.

The PSO has now been a very well-known and promising optimization algorithm in metaheuristic algorithms. It has been applied to many non-linear, complex, and multi-modal optimization problems to give brilliant results. There are almost more than two dozen of its variants as discussed in reference [23]. The PSO has two equations as shown in Eqs 42 and 43.

$$\begin{array}{c} v_{i}^{n+1}=wv_{i}^{n}+\left(\alpha \left(n\right)\epsilon_{1}\times \left(g^{*n}-x_{i}^{n}\right)\right)+\left(\beta \left(n\right)\epsilon_{2}\times \left(p_{i}^{n}-x_{i}^{n}\right)\right) \end{array}$$ (42)

$$\begin{array}{c} x_{i}^{n+1}=x_{i}^{n}+v_{i}^{n+1} \end{array}$$ (43)

where, $v_{i}^{n+1}$ is known as velocity, in PSO terminology, that is added in current position $x_{i}^{n}$ of the particle to get the new position $x_{i}^{n+1}$ and *g* is the global best and *p* is the local best of *i*^*th*^ particle of *n*^*th*^ iteration. The acceleration coefficients are *α* and *β*, usually varied between 0 to 2.1, *w* is the inertial factor of the particles having a value in the range of 0 to 1, and *ϵ* is a random number generated in between 0 and 1, according to reference [23]. Lower values of acceleration coefficients make the particle’s trajectory smooth while higher values cause abrupt movements. The inertial factor controls the impact of the previous velocity in a new direction and is used to balance exploration and exploitation. Larger values of inertial factor result in exploration (may cause divergence of the swarm) and small values cause exploitation (decelerate the particles resulting in the convergence of swarm). The values of the tuning parameters of the PSO update equation can be taken as constant as well as varying per the number of iterations (parameter control).

It has been observed solving the CSTHTS problem that the constant values of these tuning parameters of PSO do not give good results but using the parameter control for the tuning parameters, PSO gives brilliant results. Value of *α* and *w* is linearly decreased from *α*_*max*_ = 1.95 to *α*_*min*_ = 1.8 and *w*_*max*_ = 0.001 to *w*_*min*_ = 0, respectively and *β* value is sinusoidally decreased from *β*_*max*_ = 1.95 to *β*_*min*_ = 1.8 $\left(\frac{1}{2}\;\text{to}\;\frac{3}{4}\;\text{of}\;\text{wavelength}\right)$ in this research as shown in Eqs 44–46. This variant of PSO is simulated for 50 trials having 2500 iterations and population size of 500. The computational time of simulating one trial of this variant is 34 seconds.

$$\begin{array}{c} w=w_{max}-(\left(w_{max}-w_{min}\right)\times \frac{Iteration_{current}}{Iteration_{total}}) \end{array}$$ (44)

$$\begin{array}{c} \alpha =\alpha_{max}-(\left(\alpha_{max}-\alpha_{min}\right)\times \frac{Iteration_{current}}{Iteration_{total}}) \end{array}$$ (45)

$$\begin{array}{c} \beta =\beta_{max}-(\left(\beta_{max}-\beta_{min}\right)\times \text{sin}(\frac{\pi }{2}\times \frac{Iteration_{current}}{Iteration_{total}})) \end{array}$$ (46)

## Results

**Fig 1:**
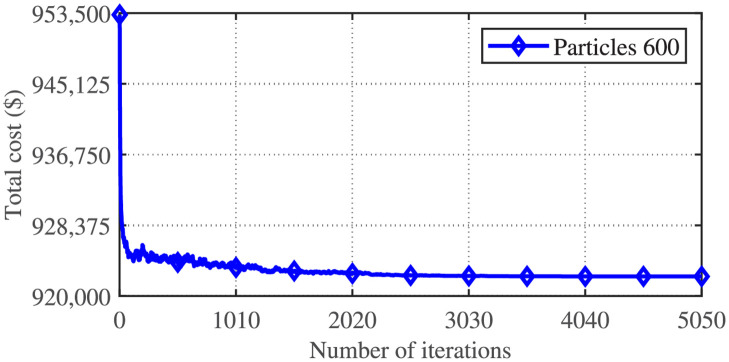
Convergence characteristics of APSO variant-16 for the CSTHTS problem.

The test system selected in this article is simulated on MATLAB run on Intel(R) Core (TM) i3-3220 CPU at 3.30 GHz system having 8 GB RAM. The minimum cost achieved by the sixteen variants of the APSO algorithm is shown in Table 2. It can be seen that APSO variant-16 gives the best results compared with the remaining fifteen variants of APSO in terms of minimum, maximum, average, and standard deviation of cost achieved for the CSTHTS problem, considered in this article. Fig 1 shows the convergence behavior of APSO variant-16 applied to the CSTHTS problem.

**Table 2:** Comparison of cost obtained by implementing 16 variants of APSO.

Variant. no	Minimum ($)	Maximum ($)	Average ($)	Std. deviation ($)
1	922329.424646790	922339.476833197	922332.983292443	2.139597945750570
2	922329.020515838	922334.796166299	922331.411276286	1.361088807162620
3	922328.309587081	922337.839284874	922332.355857353	2.032272933703020
4	922327.913035623	**922480.083143976**	**922337.100608413**	29.226889763077900
5	922327.709299195	922334.015275867	922331.044136979	1.560290880362930
6	922327.089041942	922333.380035461	922330.347735337	1.538656482837650
7	922326.308678703	922333.251583416	922329.538070115	1.654475638133620
8	922325.765024078	922332.480979352	922328.474060958	1.470186548807700
9	922325.698801677	922331.570833448	922328.615632545	1.420360604616600
10	922325.066543891	922331.353186634	922327.228495890	1.410966866808890
11	922324.774597344	922332.102952866	922327.486961129	1.543005745185750
12	922324.640787209	922475.393031006	922332.193065414	**29.3062176480051**
13	922324.623452044	922331.144321152	922327.026445323	1.466999848444430
14	922324.397350414	922328.697472803	922326.307872664	1.057550967469090
15	922324.381682261	922331.002347075	922326.720440887	1.247227232472900
16	**922323.966688770**	**922328.357852968**	**922326.214371057**	**0.975084114589519**

**Fig 2:**
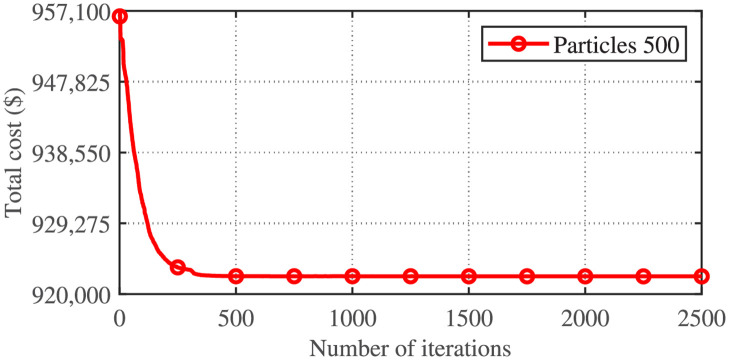
Convergence characteristics of Improved PSO for the CSTHTS problem.

The PSO variant (Improved PSO) applied to the CSTHTS problem gives the best-achieved minimum result so far compared to the sixteen variants of APSO discussed in this article as well as results reported in the literature, according to the reference [24]. However, the maximum, average, and standard deviation of the cost obtained by implementing Improved PSO are higher. Table 3 shows the comparison of the cost obtained by implementing Improved PSO with APSO variant-16 for 50 trials. Tables 4 and 5 show the power flow and the minimum cost obtained by implementing APSO variant-16 on the CSTHTS problem and Tables 6 and 7 show the power flow and the minimum cost obtained by implementing Improved PSO on the CSTHTS problem. It can be seen that no constraint of the power system under consideration has been violated. Fig 2 shows the convergence behavior of Improved PSO applied to the CSTHTS problem.

**Table 3:** Comparison of cost obtained from APSO variant-16 and Improved PSO.

Algorithm applied	Minimum ($)	Maximum (S)	Average ($)	Standard. deviation ($)
**Improved PSO**	922320.6528	923506.5519	922623.8742	306.8276
**APSO variant-16**	922323.9667	922328.3579	922326.2144	0.9751

**Table 4:** Power flow and cost optimization with APSO variant-16 of APSO algorithm (a).

Interval	Volume 1 (acre-ft)	Volume 2 (acre-ft)	Volume 3 (acre-ft)	Volume 4 (acre-ft)	Q1 (acre-ft/hr)	Q2 (acre-ft/hr)	Q3 (acre-ft/hr)	Q4 (acre-ft/hr)
1	99.9798205884863	80.6295627173402	148.1000000006460	109.7999999999290	10.0201794115137	7.3704372826598	29.9999999993541	13.0000000000705
2	98.6704373014632	82.5446664558474	126.3000000038180	99.1999999999295	10.3093832870232	6.0848962614929	29.9999999968281	13.0000000000000
3	97.6137183402722	85.5424805443407	110.3201794155210	87.7999999996790	9.0567189611910	6.0021859115067	29.9999999998107	13.0000000002505
4	96.1281719803436	88.5246905466874	100.0000000088240	74.7999999971477	8.4855463599285	6.0177899976533	29.9999999763793	13.0000000025313
5	93.9396975092428	90.5184668394184	100.0000000000000	91.7999999965018	8.1884744711009	6.0062237072691	18.1416152315082	13.0000000000000
6	92.8625466707071	91.2813975507861	100.3632666319620	108.7999999922190	8.0771508385357	6.2370692886323	18.1244656394732	13.0000000011111
7	92.6036492935517	90.6686485222112	100.6608003372080	125.7999999920300	8.2588973771554	6.6127490285749	16.9087307635081	13.0000000000000
8	93.1314608617446	90.4166698784866	100.8277635198310	142.7999999684090	8.4721884318071	7.2519786437246	15.9164113631816	13.0000000000000
9	94.5044900876941	90.6147973640683	101.2420268752360	147.9416151948800	8.6269707740506	7.8018725144182	15.0817033103828	13.0000000050375
10	96.7989686212378	91.5450872079506	102.2239787014280	153.0660808129550	8.7055214664562	8.0697101561177	15.1029856341898	13.0000000213980
11	100.1438193392990	92.4209781019504	103.6949710984180	156.9748115700940	8.6551492819389	8.1241091060003	15.4079570207855	13.0000000063691
12	101.4889700720650	91.9743682998747	106.5173256713770	159.6880709909370	8.6548492672344	8.4466098020757	15.6850394079157	13.2031519423380
13	103.9944604714250	91.4543293736483	111.1297033635380	160.0000000000000	8.4945096006391	8.5200389262263	16.1124817458954	14.7697743013202
14	107.5041088051440	91.7773934899289	114.3983015499160	160.0000000000000	8.4903516662816	8.6769358837194	16.5103601868567	15.1029856341898
15	110.2065751941360	91.9519804025535	117.3887754011060	160.0000000000000	8.2975336110082	8.8254130873753	16.9506455515242	15.4079570207855
16	112.1422050263140	90.9787414809159	119.0504355642790	160.0000000000000	8.0643701678218	8.9732389216377	17.3487304293351	15.6850394079157
17	113.1596182176280	88.6572608772882	121.2477916207790	160.0000000000000	7.9825868086858	9.3214806036276	16.7771134382281	16.1124817458954
18	113.3677193482690	85.0107780805137	124.3467785141440	160.0000000000000	7.7918988693586	9.6464827967745	15.7907963618320	16.5103601868567
19	112.6683469905360	81.7410914816218	127.5881553774080	160.0000000000000	7.6993723577339	10.2696865988919	14.7144488670595	16.9506455515242
20	111.0413044194700	78.7998570774155	132.1270980523210	159.9999999867630	7.6270425710656	10.9412344042063	13.5744367980732	17.3487304425720
21	110.5291944346650	76.0853284538525	141.4729531936820	158.5241252806990	7.5121099848047	11.7145286235630	10.0000000131476	18.2529881442926
22	111.0667497180990	75.5417249217951	151.3696823636390	154.4941924250280	7.4624447165661	9.5436035320574	10.0000000000000	19.8207292175031
23	115.0001417619580	73.1958133379682	160.8230267509600	148.2670505986000	5.0666079561408	10.3459115838269	10.0000000016902	20.9415906934872
24	120.0000000000000	70.0000000000000	170.0000000000000	140.0000000000000	5.0001417619583	11.1958133379681	10.0000000910893	21.8414873966730

**Table 5:** Power flow and cost optimization with APSO variant-16 of APSO algorithm (b).

Interval	Power Demand (MW)	P Hydel 1 (MW)	P Hydel 2 (MW)	P Hydel 3 (MW)	P Hydel 4 (MW)	P Thermal (MW)	Individual Cost ($)	Total Cost ($)
1	1370	86.0853757506495	58.5494734528380	0.0000000000000	200.0936800005480	1025.2714707959600	26787.5754169388000	922323.96668877
2	1390	86.8845541515415	51.0792936335313	0.0000000000000	187.7552799999160	1064.2808722150100	27699.5802964537000	
3	1360	80.4717503929585	52.1628874276380	0.0000000000000	173.7332800016760	1053.6320821777300	27450.0171070007000	
4	1290	76.7893045427112	53.8673748783840	0.0000000000000	156.7916800163200	1002.5516405625800	26259.2110827911000	
5	1290	74.2820885406850	54.8085015157776	25.4416841349794	178.7420799956610	956.7256458128970	25199.7803223198000	
6	1410	73.2302898980260	56.8533933205558	25.6854528727951	198.9584800010070	1055.2723839076200	27488.4293795024000	
7	1650	74.2116065290061	59.1751601344916	30.2064010144491	217.4408799917410	1268.9659523303100	32584.6954610891000	
8	2000	75.6356975786274	63.3262421194186	33.1973328487007	234.1892799704880	1593.6514474827700	40677.5576637969000	
9	2240	77.0134163339801	66.9182039116305	35.3718647227097	238.9132683446640	1821.7832466870200	46616.0267322097000	
10	2320	78.2705183505673	69.0467021311822	35.7543917845499	243.4636756652250	1893.4647120684800	48524.9397034118000	
11	2230	79.0999504693796	69.8346297868349	35.7184078137597	246.8286131844660	1798.5183987455600	46000.8901171673000	
12	2310	79.5189768384181	71.5059441526975	36.2828007362804	251.1803291395480	1871.5119491330600	47938.1433748503000	
13	2230	79.3133967113409	71.6533255425818	37.1647229335630	266.5569329974850	1775.3116218150300	45389.4458479515000	
14	2200	80.2235349500763	72.7231931348842	37.4518948811866	269.5756425477280	1740.0257344861200	44463.8732154815000	
15	2130	79.6668750415002	73.6523535091472	37.4042796054181	272.2781793589340	1666.9983124850000	42564.1343473677000	
16	2270	78.5693945571167	73.9429141760311	36.8406475332985	274.6835789723760	1605.9634647611800	40992.7358237101000	
17	2130	78.2241914264072	74.5121573809510	39.4759369074557	278.3009245011420	1659.4867897840400	42369.9391747891000	
18	2140	76.9712741584860	74.0309800415974	43.1854243363818	281.5662806382940	1664.2460408252400	42492.9537526496000	
19	2240	76.2063050488255	74.9723348567708	46.3859241966401	285.0652672737670	1757.3701686240000	44918.2070567198000	
20	2280	75.3962360406965	75.9552265297222	49.2273071246866	288.1254228249670	1791.2958074799300	45810.3608434050000	
21	2240	74.4950924410213	77.0698896459225	50.5929833465604	293.2692433575980	1744.5727912089000	44582.8660388636000	
22	2120	74.2499560681120	67.4455847273419	52.7846316487206	299.2485385274870	1626.2712890283400	41513.9253603799000	
23	1850	55.3192724051720	69.5466741372173	54.5854149677788	298.6204921994740	1371.9281462903600	35105.3940859423000	
24	1590	55.0213325539675	70.7119598259763	56.0600001712479	293.2559686861480	1114.9507387626600	28893.2844839779000	

**Table 6:** Power flow and cost optimization with Improved PSO algorithm (a).

Interval	Volume 1(acre-ft)	Volume 2(acre-ft)	Volume 3(acre-ft)	Volume 4(acre-ft)	Q1 (acre-ft/hr)	Q2 (acre-ft/hr)	Q3 (acre-ft/hr)	Q4 (acre-ft/hr)
1	99.9745088497391	80.7031236589509	148.1000043665080	109.8000000000000	10.0254911502609	7.2968763410491	29.9999956334919	13.0000000000000
2	98.5964439895096	82.5164518987279	126.3000045939140	99.2000000000000	10.3780648602295	6.1866717602231	29.9999997725943	13.0000000000000
3	97.5411162975255	85.5164511246727	110.3254958510000	87.8000000000000	9.0553276919841	6.0000007740552	29.9999998931746	13.0000000000000
4	96.0637321869161	88.5164511246727	100.0004370522790	74.7999297414654	8.4773841106095	6.0000000000000	30.0000000000000	13.0000702585346
5	93.8481458448060	90.5085437594022	100.0004292030810	91.7999253749573	8.2155863421101	6.0079073652705	18.2420073014046	13.0000000000000
6	92.7876266221728	91.3398603745664	100.5817730052300	108.7999251475520	8.0605192226333	6.1686833848358	17.8960410825158	13.0000000000000
7	92.6429440416374	90.6759887210104	100.9634691240370	125.7999250407260	8.1446825805354	6.6638716535560	16.8338902233027	13.0000000000000
8	93.2111582099707	90.4116928390378	101.1792366114690	142.7999250407260	8.4317858316667	7.2642958819727	15.8526591004721	13.0000000000000
9	94.6006874964342	90.6037883858837	101.4549113094520	148.0419323421310	8.6104707135365	7.8079044531541	15.0376912673881	13.0000000000000
10	96.8764511641940	91.5384598534448	102.5195601211100	152.9379734246470	8.7242363322402	8.0653285324389	15.0310086735645	13.0000000000000
11	100.2148209184630	92.4174209966738	104.0103832643630	156.7718636479490	8.6616302457307	8.1210388567710	15.3839434522568	13.0000000000000
12	101.5352415905500	91.9374709489402	106.9120589561320	159.4165456969020	8.6795793279132	8.4799500477337	15.6304650936254	13.2079770515191
13	103.9555233887080	91.4577169586805	111.4970601936320	160.0000000000000	8.5797182018415	8.4797539902597	16.1419575406691	14.4542369642903
14	107.5146790491930	91.7486678753866	114.7498700280320	160.0000000000000	8.4408443395159	8.7090490832939	16.5478083502840	15.0310086735645
15	110.2076786458700	91.9757396543676	117.8068914659690	159.9999979745870	8.3070004033227	8.7729282210190	17.0026468116381	15.3839454776701
16	112.1265085170890	90.9978384908390	119.2832381234220	159.9999985247360	8.0811701287810	8.9779011635286	17.4442516723229	15.6304645434761
17	113.1515508266880	88.6488527366307	121.5321692056730	159.9999986294120	7.9749576904011	9.3489857542083	16.7671184043654	16.1419574359935
18	113.3587456572800	84.9515013195247	124.4537119507820	159.9999990539580	7.7928051694077	9.6973514171060	15.9325556046917	16.5478079257377
19	112.6596417302290	81.6811364967629	127.5702600396460	159.9999989792820	7.6991039270512	10.2703648227618	14.8363107650656	17.0026468863141
20	111.0347818294700	78.6981082898714	132.0819017282930	159.9999995170430	7.6248599007593	10.9830282068915	13.6301492349684	17.4442511345622
21	110.5077676239340	76.0278333444615	141.4783570724500	158.2907863678050	7.5270142055359	11.6702749454099	10.0000000000000	18.4763315536031
22	111.0616665067920	75.4308627575316	151.3735816678260	154.4384730715240	7.4461011171413	9.5969705869300	10.0000001281457	19.7848689009728
23	115.0036822734890	73.1732607062298	160.8836240802530	148.3609128012180	5.0579842333037	10.2576020513018	10.0000000000000	20.9138710353714
24	120.0000000000000	70.0000000000000	170.0000000000000	140.0000000000000	5.0036822734888	11.1732607062298	10.0000001428044	21.9910620361864

**Table 7:** Power flow and cost optimization with Improved PSO algorithm (b).

Interval	Power Demand (MW)	P Hydel 1 (MW)	P Hydel 2 (MW)	P Hydel 3 (MW)	P Hydel 4 (MW)	P Thermal (MW)	Individual Cost ($)	Total Cost ($)
1	1370	86.1077873385179	58.1297989420904	0.0000000000000	200.0936800000000	1025.6687337193900	26796.8323900714000	922320.652836039
2	1390	87.1495933890909	51.7813383509611	0.0000000000000	187.7552800000000	1063.3137882599500	27676.8971591984000	
3	1360	80.4387903492418	52.1329867913553	0.0000000000000	173.7332800000000	1053.6949428594000	27451.4889681155000	
4	1290	76.7199267139793	53.7345864045211	0.0000000000000	156.7921449271490	1002.7533419543500	26263.8926951248000	
5	1290	74.4100674719813	54.8156928091766	25.0388095941166	178.7419874500050	956.9934426747200	25205.9469979995000	
6	1410	73.1032388209212	56.3939563852472	26.6812238671385	198.9583948029260	1054.8631861237700	27478.8458564546000	
7	1650	73.5534344584183	59.5305097675237	30.5870622563970	217.4408023271840	1268.8881911904800	32582.8077543424000	
8	2000	75.4351471670771	63.4037048284998	33.5220235927895	234.1892099730300	1593.4499144386000	40672.4036168700000	
9	2240	76.9561145614169	66.9495994426618	35.5579640224001	239.0038596299800	1821.5324623435400	46609.3842997386000	
10	2320	78.4019986770961	69.0167657419542	36.0341364793684	243.3518392578280	1893.1952598437500	48517.7255727898000	
11	2230	79.1596974330591	69.8143106312195	35.9098871814732	246.6561560985240	1798.4599486557200	45999.3473880274000	
12	2310	79.6757631631658	71.6797596511249	36.5826502459366	251.0012875673610	1871.0605393724100	47926.0974399435000	
13	2230	79.8092153559049	71.4226200593951	37.2416918776032	263.6348816576430	1777.8915910494500	45457.3155671982000	
14	2200	79.9234902261820	72.8895694388376	37.4923578125550	268.9294001588230	1740.7651823636000	44483.2183416399000	
15	2130	79.7270681324400	73.3711821253855	37.4182118529212	272.0674880148520	1667.4160498744000	42574.9407243460000	
16	2270	78.6768437868693	73.9789017034147	36.6243003735259	274.2135683338530	1606.5063858023400	41006.6481426523000	
17	2130	78.1716099838292	74.6511729493625	39.6172771625416	278.5461941138890	1659.0137457903800	42357.7171366181000	
18	2140	76.9758582374261	74.2480007345386	42.9028563025568	281.8685567071280	1664.0047280183500	42486.7142476872000	
19	2240	76.2028425181839	74.9370371715421	46.1872514124507	285.4705896451340	1757.2022792526900	44913.8034620733000	
20	2280	75.3798414894861	76.0581229919429	49.1677147151055	288.8450920557300	1790.5492287477400	45790.6782730947000	
21	2240	74.5951828596326	76.8688068109337	50.5942655481191	294.5783507648080	1743.3633940165100	44551.2090123104000	
22	2120	74.1334320008320	67.6312071285805	52.7854335951837	298.9788318003670	1626.4710954750400	41519.0614819522000	
23	1850	55.2402577055474	69.1406452468902	54.5960358198607	298.5857539846320	1372.4373072430700	35117.9646236921000	
24	1590	55.0546076759563	70.6253740079106	56.0600002684722	293.8829720647810	1114.3770459828800	28879.7116840984000	

Proper statistical tests are required to establish the superiority of one algorithm over the other, according to reference [24]. So, non-parametric Mann-Whitney U test and parametric Independent samples t-test have been performed using SPSS software on the data sets obtained by running APSO variant-16 and Improved PSO for 50 trials. Results of the Mann-Whitney U test have been presented in Tables 8 and 9 and results of the Independent samples t-test have been presented in Tables 10 and 11. It can be seen that APSO variant-16 is statistically different and better than Improved PSO thus superiority of APSO variant-16 over Improved PSO has been established statistically. Table 12 shows the comparison of results achieved by implementing APSO variant-16 and Improved PSO on the CSTHTS problem with the reported results in the literature presented in reference [24].

**Table 8:** Non-parametric Mann Whitney U test for the CSTHTS problem.

Test Statistics	
**Mann-Whitney U**	750.000
**Wilcoxon W**	2025.000
**Z**	-3.447
**Asymp. Sig. (2-tailed)**	0.001

**Table 9:** Rank statistics of non-parametric Mann Whitney U test for the CSTHTS problem.

Ranks			
Group	N	Mean Rank	Sum of Ranks
**APSO variant-16**	50	40.50	2025.00
**Improved PSO**	50	60.50	3025.00
**Total**	100		

**Table 10:** Group statistics of independent sample t-test for the CSTHTS problem.

Group Statistics				
Group	N	Mean	Std. Deviation	Std. Error Mean
**APSO variant-16**	50	922326.2144	0.9750841146	0.1378977179
**Improved PSO**	50	922623.8742	306.8276371	43.39198057

**Table 11:** Independent sample t-test for equality of means for the CSTHTS problem.

Independent samples test	Levene’s test for equality of variances		t-test for equality of means						
	F	Sig.	t	df	Sig. (2-tailed)	Means difference	Std. error difference	95% confidence interval of the difference	
								Lower	Upper
**Equal variances assumed**	83.742	.000	-6.860	98	.000	-297.659782	43.39219969	-383.770190	-211.549373
**Equal variances not assumed**			-6.860	49.001	.000	-297.659782	43.39219969	-384.859627	-210.459936

**Table 12:** Comparison of cost obtained by implementation of different algorithms on the CSTHTS problem.

Algorithm	Minimum cost ($)	Average cost ($)	Maximum cost ($)	Computation time (sec)
FEP [7]	930267.92	930897.44	931396.81	NA
CEP [7]	930166.25	930373.23	930927.01	NA
IFEP [7]	930129.82	930290.13	930881.92	NA
GA [2]	932734	936969	939734	NA
IPSO [8]	922553.49	NA	NA	NA
APSO [9]	926151.54	NA	NA	NA
MAPSO [9]	922421.66	922544	923508	NA
DE [10]	923991.08	NA	NA	NA
BCGA [11]	926922.71	927815.35	929451.09	NA
RCGA [11]	925940.03	926120.26	926538.81	NA
EGA [12]	934727	936058	937339	NA
PSO [12]	928878	933085	938012	NA
EPSO [12]	922904	923527	924808	NA
MDE [13]	922555.44	NA	NA	NA
TLBO [14]	922373.39	922462.24	922873.81	NA
RCGA-AFSA [15]	922340	922362	922346	NA
MFO [16]	924455	925431	924836	NA
GWO [16]	924259	925210	924784	NA
PSO-ALNS [16]	923542	924025	923755	NA
CGWO-DA [16]	923259	923711	923444	67
SPPSO [17]	922336.31	NA	NA	16
Canonical APSO [24]	922615.3048	923322.9877	924967.5195	80
Improved APSO [24]	922335.6037	922351.7587	922443.6	100
**Proposed APSO variant-16**	922323.9667	922326.2144	922328.3579	45
**Proposed Improved PSO**	922320.6528	922623.8742	923506.5519	34

## Discussion

Sixteen variants of APSO and one variant of PSO (Improved PSO) have been applied to solve the CSTHTS problem. All of them have been run for 50 trials and give better results than the so-far best result of the CSTHTS problem which is 922335.6037 $, as found in literature, according to reference [24]. It can be noticed that the APSO variant-16 is the same as that adopted by the reference [24]. However, it was observed that by selecting the maximum and minimum values of both *α* and *β* as 0.81 and 0.62 respectively, the results were significantly improved, as presented in Table 2.

The minimum cost achieved by the sixteen variants and their standard deviations is improved as compared to the improved APSO-3 results previously achieved and reported in reference [24]. It can be observed that PSO in its improved version has achieved the so far best result for the CSTHTS problem under consideration in this article, even better than APSO variant-16. However, it can be observed in Table 3, that the standard deviation achieved by APSO variant-16 is far better than Improved PSO.

Tables 8 and 9 give the Mann Whitney U test and Tables 10 and 11 give the Independent samples t-test for the CSTHTS problem, between APSO variant-16 and Improved PSO. Although, Improved PSO has given the nearest approximation to the global optimum solution, however, the sixteen variants of APSO discussed are statistically far superior as compared to Improved PSO, as their standard deviations are lesser, and their minimum is very near to the best value achieved by Improved PSO. Also, the highest values of best cost achieved by sixteen variants of APSO are far lesser than the highest value of best cost achieved by Improved PSO. So, it can be concluded that sixteen variants of APSO are statistically different and superior in performance for the greatest number of trials as compared to Improved PSO.

**Fig 3:**
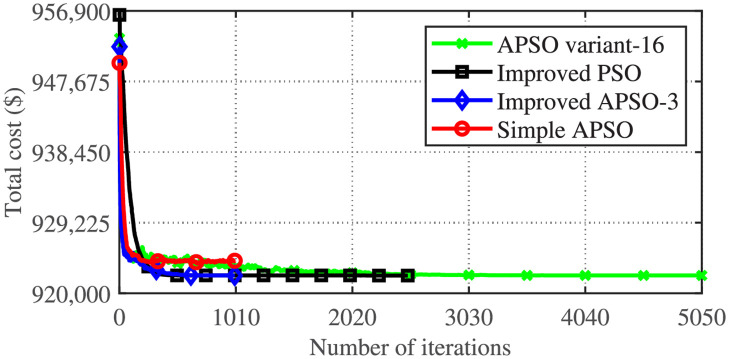
Comparison of the convergence behavior of the best trials of four metaheuristic algorithms on the CSTHTS problem.

**Fig 4:**
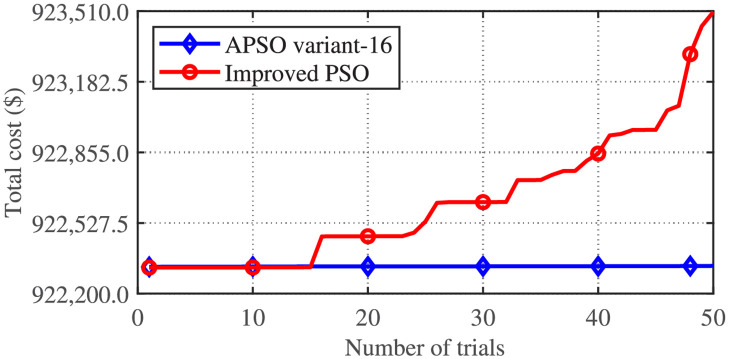
Comparison of minimum costs achieved by APSO variant-16 and Improved PSO for 50 trials for the CSTHTS problem.

Fig 3 gives the comparison of the convergence behavior of the best trial of the four metaheuristic algorithms applied to the CSTHTS problem. Improved PSO and two variants of APSO (APSO variant-16 and the variant applied in reference [24]) give almost the same convergence characteristics for their best trial. Simple APSO, however, shows inferior performance. Fig 4 gives the comparison of the minimum costs achieved by the APSO variant-16 and Improved PSO, for 50 trials. Though the nearest approximation to the minimum value is achieved by Improved PSO, however, it is not much different from the minimum achieved by the APSO variant-16. Moreover, the APSO variant-16 has statistically outperformed Improved PSO.

There is a flaw in this benchmark CSTHTS problem that for some combinations of discharge rates and volumes of reservoirs, the values of hydel powers come negative, according to reference [27]. Since this is a non-pumped storage hydrothermal scheduling problem, these negative values of hydel power mean nothing and therefore are fixed to zero value, according to reference [28], and the power plant may be simply shut down during these intervals and the water may be discharged out just as spillage water (spillage water does not produce hydel power). This issue of the problem has been adopted previously as it is and all the research articles related to this CSTHTS problem, as mentioned in the literature, till date adopt the same mathematical model, and thus the optimal scheduling is taken as it is.

## Conclusion

An important case of the CSTHTS problem has been solved by implementing sixteen newly proposed variants of the APSO algorithm and one newly proposed variant of the PSO algorithm. The results achieved are better than the reported so far best results achieved by the other algorithms, as found in the literature, according to reference [24]. The proposed algorithms, not only helped to achieve the so far best approximation of the global optimum solution, but also they achieved it persistently in every repeated trial, i.e., by maintaining low standard deviation, and in very economical computation time.

It can be concluded that APSO in its canonical as well as in improved form gives good approximates to the global best solution and is easy to program. PSO also gives good approximates to the global optimum solution but on the statistical basis, it has been established that statistically, the improved variants of APSO outperformed the improved PSO variant on the CSTHTS problem, though, the improved PSO helped to achieve the so far best approximate of the global optimum solution.

Furthermore, it has been established that true statistical hypothesis testing is mandatory to validate the superiority of one type of metaheuristic algorithm over the other type of metaheuristic algorithm for a given short-term hydrothermal scheduling problem.

## Abbreviations

*APSO*: Accelerated particle swarm optimization
*CSTHTS*: Cascaded short-term hydrothermal scheduling
*F*_(*m*,*i*)_: Fuel cost at *m*^*th*^ scheduling interval of *i*^*th*^ thermal generation unit
$I_{hyd_{j,m}}$: Inflow for the *j*^*th*^ reservoir at *m*^*th*^ scheduling interval
*N*: Total number of the scheduling interval
*N*_*h*_: Total number of hydel generation units
*N*_*s*_: Total number of thermal generation units
*P*_*d*_: Load demand
*P*_*l*_: Transmission losses
*PSO*: Particle swarm optimization
$P_{th_{i}}^{min}$: Minimum thermal power generated by the *i*^*th*^ thermal generation unit.
$P_{th_{i,m}}$: Thermal power at *m*^*th*^ scheduling interval of *i*^*th*^ thermal generation unit
$P_{hyd_{j,m}}$: Hydel power at *m*^*th*^ scheduling interval of *j*^*th*^ thermal generation unit
$P_{th_{i}}^{max}$: Maximum thermal power generated by the *i*^*th*^ thermal generation unit.
$P_{hyd_{j}}^{min}$: Minimum hydel power generated by the *j*^*th*^ hydel generation unit
$P_{hyd_{j}}^{max}$: Maximum hydel power generated by the *j*^*th*^ hydel generation unit
$Q_{hyd_{j,m}}$: The discharge rate of the *j*^*th*^ reservoir at *m*^*th*^ scheduling interval
$Q_{hyd_{j}}^{min}$: Minimum allowable value of the discharge rate of the *j*^*th*^ reservoir based hydel generation unit at *m*^*th*^ scheduling interval
$Q_{hyd_{j}}^{max}$: Maximum allowable value of the discharge rate of the *j*^*th*^ reservoir based hydel generation unit at *m*^*th*^ scheduling interval
$Q_{hyd_{j,total}}$: The total value of the allowed discharge rate of the *j*^*th*^ reservoir based hydel generation unit for a total period of *N* scheduling interval
*R*_*u*,*j*_: Number of the upstream reservoirs present for the *j*^*th*^ reservoir
$S_{hyd_{j,m}}$: Spillage of the *j*^*th*^ reservoir at *m*^*th*^ scheduling interval
*t*: Water transport delay
$V_{hyd_{j,m}}$: Volume of the *j*^*th*^ reservoir at *m*^*th*^ scheduling interval
$V_{hyd_{j}}^{min}$: Minimum allowable value of the volume of the *j*^*th*^ reservoir based hydel generation unit at *m*^*th*^ scheduling interval
$V_{hyd_{j}}^{max}$: Maximum allowable value of the *j*^*th*^ reservoir based hydel generation unit at *m*^*th*^ scheduling interval
