# Supplementary material for: Impact of parameter control on the performance of APSO and PSO algorithms for the CSTHTS problem: An improvement in algorithmic structure and results
Source: PLoS One. 2021 Dec 17;16(12):e0261562. doi: 10.1371/journal.pone.0261562 (PMC8682890; doi:10.1371/journal.pone.0261562)
Supplement: Attachment — Submitted filename: Review Comments to Authors.docx [file pone.0261562.s001.docx]

**Comments to the Author**

The authors presented accelerated particle swarm optimization (APSO) algorithm for solving cascaded hydrothermal scheduling problem. Although the research article has presented valuable contributions but require some minor changes in the manuscript.

1. It is suggested to organize as follows the complete manuscript (1) introduction (2) formulation (3) proposed technique (4) simulation results (5) conclusions.
2. What is the feasibility analysis of all the variants in terms cost calculations of the given problem? It is suggested to mention a justification of variants as mentioned in table 2 and provide the comment on each variant.
3. What is the significance of inertia weight parameters? Is there any influence on the variation of alpha and beta parameters?
4. On what analysis basis the improved PSO is better as compared to other variants of PSO?
5. Check the standard deviation of the proposed techniques
6. Why the hydro generation is zero in table 5 for third hydro plant even the discharge shown in table 4? Is there any technical reason or the calculations mismatch?
