# Supplementary material for: Impact of parameter control on the performance of APSO and PSO algorithms for the CSTHTS problem: An improvement in algorithmic structure and results
Source: PLoS One. 2021 Dec 17;16(12):e0261562. doi: 10.1371/journal.pone.0261562 (PMC8682890; doi:10.1371/journal.pone.0261562)
Supplement: Attachment — Submitted filename: Response to Reviewers.docx [file pone.0261562.s002.docx]

**Reviewer#1:** In this paper, the authors analyze the impact of parameter control on the performance of APSO and PSO algorithms for the CSTHTS problem. Here, there are some concerns of this reviewer:

**Concern#1:** Introduction can be improved by having four clear and concise subsections on the motivation of your research; background and related works; list of key contributions/future trends; and organization of the paper.

Comments: We highly thank the worthy reviewer for suggesting an update in the introduction section.

Action taken: We have added two paragraphs at the end of the introduction section to meet the concerns 1 of the worthy reviewer.

**Concern#2:** The novelty and main contributions of this paper should be further summarized and clearly demonstrated. This reviewer suggests the authors exactly mention what is new compared with existing approaches and why the proposed approach is needed to be used instead of the existing methods. Especially, the comparison of this paper with the authors’ previous works [R1, R2] must be performed to highlight the contributions of this work.

[R1] Fakhar MS, Kashif SAR, Ain NU, Hussain HZ, Rasool A, Sajjad IA. Statistical performances evaluation of APSO and improved APSO for short term hydrothermal scheduling problem. Applied Sciences. 2019;9(12):2440.

[R2] Liaquat S, Fakhar MS, Kashif SAR, Rasool A, Saleem O, Padmanaban S. Performance Analysis of APSO and Firefly Algorithm for Short Term Optimal Scheduling of Multi-Generation Hybrid Energy System. IEEE Access. 2020;8:177549–177569.

Comments: We thank the worthy reviewer for his deep insight into the paper and relevant literature and then for his concern.

Action taken: We have added in the introduction section the motivation behind writing this research article and also presented the novelty in the paper. We would respectfully like to add that the references [R1] and [R2] by the authors have a different scope of research work as those articles discuss non cascaded short term hydrothermal scheduling cases and combined solar-hydrothermal scheduling (for non-cascaded hydrothermal systems) cases, which are totally different in structure problems, as compared to the cascaded short term hydrothermal scheduling problem.

**Concern#3:** The authors should introduce more about the references, not only mentioning them.

Comments: Thank you very much worthy reviewer for the concern.

Action taken: We have overall presented the details of the results in tabular data in the results section of the paper. We have presented an overall view of the lack of previous research articles which we think sufficiently serve the purpose. A detailed discussion on several aspects, which in our opinion is out of the scope of this technical article, is already discussed in a detailed review article written by the authors, as reference [19] in the manuscript.

**Concern#4:** In this study, how to handle the problem of premature convergence in the improved variant of the Accelerated PSO (APSO) algorithm? Use of mutation strategy together with dynamic monitoring mechanism of diversity has been proven as an effective method reported in literature like [[doi.org/10.1155/2015/529724](http://doi.org/10.1155/2015/529724)]. Please discuss this.

Comments: We highly regard this in-depth analysis and response of the worthy reviewer.

Action taken: We have added a paragraph in the introduction section regarding the suggested research article and appreciated the work presented in this reference. However, we think that for this very paper, the results achieved by the improved variants of PSO and PSO are already good that this very research needs not to have an addition of mutation strategies and extreme learning machines in the suggested improvements of the proposed algorithms. However, in the future, for other types of power system scheduling problems, the concept can be utilized.

**Concern#5:** The proposed method might be sensitive to the values of its main controlling parameters. How did you determine these optimal parameters?

Comments: Thank you very much for the important question. The optimal values of the tuning parameters were selected by first running the program for several trials for several sets of the random as well as the reported values of tuning parameters. Following the trend of the trials, we incorporated the values which repeatedly gave better results. It is also important to mention that the suggested ranges of values the tuning parameters if taken other than the selected sub-range, the results are still very convincing, however, the best-achieved results with such low standard deviations were obtained by selecting the ranges of tuning parameters by incorporating the method written earlier.

Action taken: Not Applicable.

**Concern#6:** The Conclusions can be improved. This reviewer strongly suggests the authors clearly explain what the significant findings are and why your paper is really important. Some of the most important quantitative results should be reported to better demonstrate the findings of the carried-out work.

Comments: Thank you very much worthy reviewer for the concern.

Action taken: We have improved the conclusion. As far as the quantitative results are concerned, we have already mentioned them in the results and discussion section.

**Concern#7:** The nomenclature should be included to improve the readability of this paper.

Comments: Thank you very much worthy reviewer for the concern.

Action taken: We have added the nomenclatures.

**Concern#8:** All references, including article titles, should be in a uniform format. Please double-check it.

Comments: Thank you very much worthy reviewer for the concern.

Action taken: We have checked the references carefully to rule out any chance of uniformity. Thanks

**Reviewer#2:** The manuscript entitled,” Impact of parameter control on the performance of APSO and PSO algorithms for the CSTHTS problem: An improvement in algorithmic structure and results” describes a study on parametric sensitivities of evolutionary algorithms which is interesting, however, apparently the study seems very close to the authors earlier works:

Fakhar, M. S., Kashif, S. A. R., Ain, N. U., Hussain, H. Z., Rasool, A., & Sajjad, I. A. (2019). Statistical performances evaluation of APSO and improved APSO for short term hydrothermal scheduling problem. Applied Sciences, 9(12), 2440.

Fakhar, M. S., Liaquat, S., Kashif, S. A. R., Rasool, A., Khizer, M., Iqbal, M. A., ... & Padmanaban, S. (2021). Conventional and metaheuristic optimization algorithms for solving short term hydrothermal scheduling problem: A review. IEEE Access.

Fakhar, M. S., Kashif, S. A. R., Liaquat, S., Rasool, A., Padmanaban, S., Iqbal, M. A., ... & Khan, B. (2021). Implementation of APSO and Improved APSO on Non-Cascaded and Cascaded Short Term Hydrothermal Scheduling. IEEE Access.

**Concern#1:** Please modify the presented work in such a way that would make it exclusive.

Comments: Thank you very much worthy reviewer for the concern.

Action taken: We have further shaped our article by adding paragraphs in the introduction section that elaborate the research gap in the previous articles that lead us to this article. The papers of the authors mentioned are different in scope than this research article as they have been discussing the details of non-cascaded short-term hydrothermal scheduling problem and also is a special case of the combined solar-hydrothermal scheduling problem, which are all together, problems of different nature. The only article of authors, that discusses the CSTHTS problem is

Fakhar, M. S., Kashif, S. A. R., Liaquat, S., Rasool, A., Padmanaban, S., Iqbal, M. A., ... & Khan, B. (2021). Implementation of APSO and Improved APSO on Non-Cascaded and Cascaded Short Term Hydrothermal Scheduling. IEEE Access.

As mentioned by the worthy reviewer. However, we have elaborated in the introduction section that how this new article is different from the previous article and can be considered as an original work with new findings by incorporating improvements in the structure of the PSO and improved APSO algorithms by utilizing the newly proposed constraints handling technique, that helped to improve the results obtained for the CSTHTS case as compared to the results of the previous work of the authors. The improvements in results were in terms of obtaining the so far best approximate to the global optimum solution while utilizing a very smaller number of particles, and in very small computational times while achieving almost zero standard deviation.

**Concern#2:** Alternatively, the present study may be published as an addendum to the above-mentioned works.

Comments: Thank you very much for the comment.

Action taken: We have already answered this concern as the answer to concern#1 of the worthy reviewer.

**Concern#3:** Please do not consider the comments as a denial of your work, the comments are only suggestions. Based on all the above comments, I would suggest a major revision and reconsideration of the presented work.

Comments: Thank you very much for the comment.

Action taken: We have not minded the concern of the worthy reviewer. We have edited and further explained our work which we hope that the worthy reviewer will consider as sufficient.

**Reviewer#3:** The authors presented accelerated particle swarm optimization (APSO) algorithm for solving cascaded hydrothermal scheduling problems. Although the research article has presented valuable contributions but requires some minor changes in the manuscript.

**Concern#1:**  It is suggested to organize as follows the complete manuscript (1) introduction (2) formulation (3) proposed technique (4) simulation results (5) conclusions.

Comments: Thank you very much for the comment.

Action taken: We have incorporated the suggestions of the worthy reviewer.

**Concern#2:** What is the feasibility analysis of all the variants in terms of cost calculations of the given problem? It is suggested to mention a justification of variants as mentioned in table 2 and provide the comment on each variant.

Comments: Thank you very much for the comment.

Action taken: We have already presented the feasibility analysis in terms of the cost of sixteen variants, however, we have given a detailed discussion on the best performing sixteenth variant by giving its detailed results. Moreover, we have given the statistical analysis of the performances of the variant sixteen of improved APSO with the best performing variant of improved PSO.

**Concern#3:** What is the significance of inertia weight parameters? Is there any influence on the variation of alpha and beta parameters?

Comments: Thank you very much for the comment. We have already presented the significance of the inertia factor and acceleration coefficients in section 3 in the following lines. We think that the following discussion sufficiently explains the roles of inertia factor and acceleration coefficients.

w is the inertial factor of the particles having a value in the range of 0 to 1 and $\in$ is a random number generated in between 0 and 1, according to reference [23]. Lower values of acceleration coefficients make the particle's trajectory smooth while higher values cause abrupt movements. The inertial factor controls the impact of the previous velocity in a new direction and is used to balance exploration and exploitation. Larger values of inertial factor result in exploration (may cause divergence of the swarm) and small values cause exploitation (decelerate the particles resulting in the convergence of swarm).

Action taken: Not Applicable.

**Concern#4:** On what analysis basis the improved PSO is better as compared to other variants of PSO?

Comments: Thank you very much for the comment. We have incorporated one improvement in PSO for the special case of the CSTHTS problem and have given its comparison with other variants of PSO, known as improved APSO. We have concluded that the suggested variant of PSO was able to find the nearest approximation to the global optimum solution however, statistically, it was weaker in performance as compared to the sixteen improved variants of APSO. Moreover, it can be seen from the literature that out of all the other variants of PSO, small population-based PSO and improved APSO performed the best for the CSTHTS case according to references [17] and [24] respectively. However, the newly proposed improved variants of both PSO and improved APSO have given better results as compared to the results of references [17] and [24].

Action taken: No action is required.

**Concern#5:** Check the standard deviation of the proposed techniques.

Comments: Thank you very much for the comment.

Action taken: The values of the standard deviations are already very low (reaching nearly 0 for the sixteenth variant of APSO) for which we have concluded that the newly proposed variant sixteen of APSO is better statistically as compared to the newly proposed PSO, though the newly proposed PSO gave the nearest approximation to the global optimum solution (PSO minimum result was 3$ cost-effective than APSO variant 16 minimum result)

**Concern#6:** Why the hydro generation is zero in table 5 for third hydro plant even the discharge shown in table 4? Is there any technical reason or the calculations mismatch?

Comments: Thank you very much for the comment. We highly appreciate the worthy reviewer for the very deep insight into the results of the paper. Indeed, the values of discharge rates and volumes are non-zero for which the corresponding values of Hydel power must be non-zero. This issue was raised in one of the comments papers, given as follows, on which there was published a reply to comment paper.

There is a flaw in this benchmark CSTHTS problem that for some combinations of discharge rates and volumes of reservoirs, the values of hydel powers come negative. Since this is a non-pumped storage hydrothermal scheduling problem, these negative values of hydel power mean nothing and therefore are fixed to zero value, and the power plant may simply shut down during these intervals and the water may be discharged out just as spillage water (spillage water does not produce hydel power). This issue of the problem has been adopted previously as it is and all the research articles related to this CSTHTS problem, as mentioned in the literature, till date adopt the same mathematical model, and thus the optimal scheduling is taken as it is. The worthy reviewer is requested to have a cross-check of the mentioned comments articles, and also the highly cited research papers published so far in this domain.

Action taken: We have added this paragraph in the discussion section of the paper so that any possible ambiguity for the readers may be avoided.
