# Supplementary material for: Impact of parameter control on the performance of APSO and PSO algorithms for the CSTHTS problem: An improvement in algorithmic structure and results
Source: PLoS One. 2021 Dec 17;16(12):e0261562. doi: 10.1371/journal.pone.0261562 (PMC8682890; doi:10.1371/journal.pone.0261562)
Supplement: Attachment — Submitted filename: Review Comments to Authors R1.docx [file pone.0261562.s003.docx]

**Comments to the Author**

The authors revised the article and the present form of manuscript is **recommended.**
